# The Extremophiles: Adaptation Mechanisms and Biotechnological Applications

**DOI:** 10.3390/biology14040412

**Published:** 2025-04-13

**Authors:** Gorji Marzban, Donatella Tesei

**Affiliations:** 1 Department of Biotechnology and Food Science (DBL), Institute of Bioprocess Science and Engineering (IBSE), BOKU University, Muthgasse 18, 1190 Vienna, Austria; 2 Department of Biotechnology and Food Science (DBL), Institute of Microbiology and Microbial Biotechnology (IMMB), BOKU University, Muthgasse 18, 1190 Vienna, Austria

**Keywords:** eukaryotic and prokaryotic extremophile, extremozymes, extreme pressure, anti-freezing proteins, cryoprotection, extreme biotechnology

## Abstract

This review provides an overview of terrestrial extremophiles, highlighting their adaptive strengths and strategies for coping with environmental challenges through the use of specialized proteins. It also explores why their unique lifestyle and ability to adapt to extreme conditions have become a major focus of research, as well as the main benefits and advancements in the study of these organisms in recent decades. This review aims to present an objective summary of the knowledge acquired and its translation into applied science and biotechnological applications.

## Simple Summary

Extremophiles are remarkable organisms capable of growing and developing in extreme environments such as volcanic areas, polar regions, deep seas, salt and acidic lakes, deserts, and even space. These organisms therefore play an important role in understanding the limits of life and expanding our knowledge of biology. By studying extremophiles, we can unlock the secrets of their enzymes, proteins, and molecular mechanisms, leading to potential applications in biotechnological and sustainable processes. Furthermore, extremophiles are increasingly being harnessed in biotechnology to produce biofunctional molecules and biopharmaceuticals within the ecosystem-friendly circular economy.

## 1. Introduction

Extremophilic organisms, which thrive in environments characterized by extreme conditions, have redefined our understanding of life’s resilience and adaptability [1]. Their presence in harsh settings, such as hot springs, deep-sea hydrothermal vents, and hypersaline bodies of water, challenges traditional beliefs about the limits of biological activity and broadens our perspective on potential habitats supporting life on Earth and beyond (see Table 1) [2,3].

Extremophiles are crucial to our comprehension of adaptive evolution and pivotal in tracing the origins of life on our planet, as their habitats closely resemble early Earth’s conditions [4]. From an evolutionary standpoint, studies on extremophiles have revealed that some of these organisms cluster near the universal ancestors on the tree of life. Hyperthermophiles, in particular, appear to be closely related to the origin of all life on Earth, making extremophiles crucial for understanding life’s origins [5]. Hence, the study of these organisms provides valuable insights into the environmental conditions and life forms that may have existed during the early stages of Earth’s history [1]. Additionally, it suggests that their molecular building blocks—such as proteins, fatty acids, and smaller molecules—must have unique adaptive properties, distinct from those of other organisms.

In addition, their significance extends to astrobiology. The ability of life to adapt and survive in harsh terrestrial conditions suggests the possibility of analogous extremophilic life forms existing on other planets, moons, or even in environments beyond our solar system [6]. Thus, investigating Earth’s extremophiles can shed light on the potential for life beyond Earth. This research has profound implications for identifying possible habitats for life elsewhere in the universe and for developing strategies for the exploration and potential colonization of such environments [6,7,8].

The study of extremophiles began in the late 19th century with the discovery of microorganisms in hot springs [1]. However, extremophile research gained significant momentum in the latter half of the 20th century [9], propelled by advancements in genetic sequencing and DNA analysis techniques that facilitated a more in-depth exploration of these organisms [10]. Unique proteins and enzymes from extremophiles have since found applications in molecular biology. Extremophilic enzymes—extremozymes—characterized by their high stability and functionality under extreme conditions, prove valuable for in vitro molecular processes requiring high temperatures or other challenging conditions [11]. The discovery of thermoresistant enzymes from extremophiles, for instance, has been instrumental in the development of fundamental techniques of DNA analytics [12].

Extremophiles span both prokaryotic and eukaryotic domains of life, encompassing both uni and multicellular organisms [13]. The main distinction between prokaryotic and eukaryotic extremophiles lies in their cellular structure, which influence the type and severity of habitats they can inhabit [1,4]. Prokaryotes, including bacteria and archaea, represent the most common and diverse group of extremophiles. These organisms thrive in a vast array of extreme environments that are inhospitable to humans, such as hot springs, deep-sea hydrothermal vents, and acidic and alkaline regions, which collectively account for approximately 75% of our planet [14]. Prokaryotes’ prevalence in such environments is largely attributed to their simpler cellular structure, genetic flexibility, and adaptability to a wide range of conditions [15] (see Table 2). Taking into consideration that proteins constitute 60 to 70% of any cell’s composition, they must play a crucial role in empowering extremophiles to withstand harsh environmental conditions.

Extremophiles have evolved various survival strategies and present a unique ability to influence their environments through nutrient cycling and energy production, thereby contributing to enhanced habitability and biological diversity [16]. As a consequence, these habitats become more hospitable to organisms with lower tolerance and adaptation capabilities, including humans [17,18]. For example, the psychrophilic algae Chlamydomonas nivalis survives in extreme cold environments by producing anti-freeze proteins (AFPs), enabling it to maintain photosynthetic activity at sub-zero temperatures [19]. Similarly, the prokaryotic bacterium Psychrobacter sp., isolated from Antarctic ice, is fully adapted to survive in very low temperatures [20]. Both organisms contribute to their ecosystems by providing organic nutrients and oxygen, which are beneficial for heterotrophic organisms.

Eukaryotes, characterized by a true nucleus and membrane-bound organelles within their cells, include several multicellular organisms typically found in moderate or stable environments. However, certain extremophilic eukaryotes exhibit unique adaptations, allowing them to survive in conditions lethal to most other organisms [21]. While multicellular eukaryotic extremophiles—such as those living near deep-sea hydrothermal vents—are relatively recent in evolutionary terms, having originated within the last 100 million years, extremophilic bacteria and archaea trace their ancient origins back several billion years, possibly to the early history of life on Earth [13]. Examples of extremophilic eukaryotes include the thermophilic fungus *Thermomyces lanuginosus*, which produces heat-resistant enzymes for survival in high temperatures, i.e., reaching up to 80 °C [22], and the halophilic algae, *Dunaliella salina* [23], found in highly saline environments such as salt pans and salt lakes. This unicellular organism has evolved specialized mechanisms to cope with the high salt concentrations, including the production of compatible solutes that help maintain cellular osmotic balance and protect against dehydration.

Extremophiles, with their remarkable ability to cope with harsh conditions, represent a reservoir of genetic diversity, offering genes and proteins essential for adaptation. For billions of years, they have significantly expanded the range of environments habitable by life, resulting in profound ecological and global implications, which, looking ahead, also include the potential applications of their macromolecules and compounds [24]. For example, enzymes and AFPs that facilitate biological processes at very low temperatures show promising applications in food and biotech industries. Proteases, amylases, and lipases, exhibiting optimal activities at cold temperatures, are of enormous interest for establishing novel bioprocessing strategies [20,25]. Due to their versatile properties, extremozymes are widely used in wastewater treatment, bioremediation, and biodegradation as part of a green chemistry approach that involves the use of various hydrolases such as esterases, catalases, peroxidases, lipases, laccases, etc. [26]. Similarly, hydrolases derived from both bacterial and fungal extremophiles are also effective in the biodegradation of plastic materials [27,28]. Moreover, enzymes from extremophiles, particularly acidophiles and thermophiles, play a role in biomining—an eco-friendly process used for the extraction of metals, also known as bioleaching [29,30]. Considering their diverse applications, including the bioremediation and biodegradation of toxic compounds and waste material, extremophilic microorganisms serve as sustainable sources of novel biomolecules and biotechnological tools that can significantly contribute to a bio-based economy. In this context, extremophiles serve as valuable evolutionary relics that can illuminate our path through the complex train of ecological challenges posed by climate change and human-based interventions and activities such as industrial energy and goods production [31].

Scientific inquiry into extremophiles is still in its early stages. This review article aims to provide a concise overview of the repertoire of extremophilic organisms and the extremoproteins that play a central role in extremotolerance and adaptation, particularly with an eye toward their biotechnological applications. The goal is to explore the potential of this largely untapped consortium of organisms to improve technological processes with a keen awareness of the need to preserve biodiversity and pursue eco-friendly interventions, both on Earth and beyond.

## 2. Extreme Conditions Triggering the Adaptation

**Figure 1:**
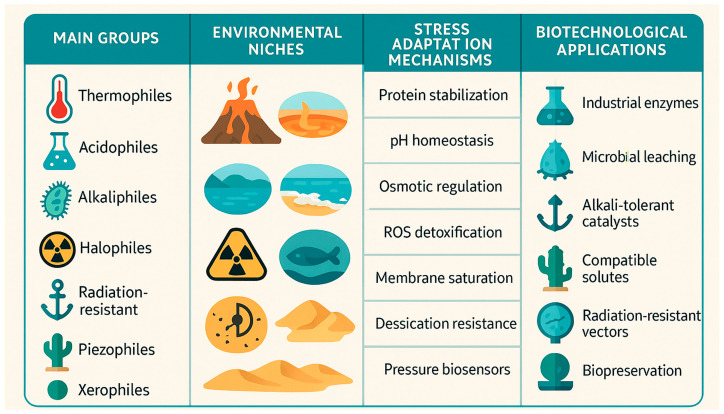
Overview of extremophiles, their environmental niches, stress adaptation mechanisms, and biotechnological applications.

Extreme environments play a significant role in driving the evolution and diversity of extremophiles (Table 1). Extremophiles are organisms that thrive in conditions considered extreme by human standards, such as high or low temperatures, high salinity, extreme pressure, high acidity or alkalinity, and high radiation levels. The mechanisms through which these environments promote the evolution and diversity of extremophiles can be illustrated by the following examples (Figure 1).

***Selective Pressure:*** Extreme conditions impose intense selective pressure, ensuring that only organisms with specific adaptative traits can survive and reproduce. Over time, these pressures can lead to the development of unique characteristics that enable survival in harsh environments. A well-known example is the *Taq* polymerase, a thermostable enzyme that remains functional at high temperatures, crucial for DNA replication in the extreme conditions of hot springs where *Thermus aquaticus* is found. This enzyme guarantees the survival of the organism and has also revolutionized molecular biology [32]. Its discovery significantly simplified the Polymerase Chain Reaction (PCR) process, by enabling the combination of the DNA denaturation and amplification step [33]. Since the advent of the Taq polymerase, other thermostable polymerases with improved properties, such as higher fidelity and greater processivity, have been discovered. These include enzymes like the Pfu polymerase, isolated from the hyperthermophilic archaeon *Pyrococcus furiosus,* which thrives in extremely hot environments, such as hydrothermal vents and various others [34].

***Genetic Mutations and Horizontal Gene Transfer:*** Harsh environments can increase mutation rates, leading to the introduction of new genetic variations [35]. Additionally, horizontal gene transfer (HGT) enables extremophiles to acquire genes from other organisms, potentially conferring advantageous traits [36,37]. HGT is especially common in prokaryotes, such as bacteria and archaea, which are frequently found in extreme environments [38]. A notable example is the radiophilic bacterium *Deinococcus radiodurans,* which has developed PprA, a DNA protection protein aiding in DNA repair and protection against radiation-induced damage, contributing to the organism’s extraordinary radiation resistance [39].

DNA protection proteins have several biotechnological applications, leveraging their ability to protect DNA from damage, degradation, or environmental stresses. These proteins are utilized across various fields of biotechnology and medicine, including (1) *Gene Cloning and Expression Systems to stabilize plasmids and other vectors*, preserving the integrity of recombinant DNA during manipulation and expression in host cells [40]; (2) *DNA Preservation in biobanking and forensic science*, in which proteins are employed to enhance the stability and longevity of DNA samples [41]; (3) *gene therapy by DNA protection proteins*, which play a critical role in gene therapy by safeguarding therapeutic genes from degradation once introduced into target cells, ensuring the efficacy and safety of gene delivery systems [42]; and (4) *Synthetic Biology* by proteins, which stabilize synthetic genetic circuits and engineered pathways, thereby improving the reliability and performance of engineered organisms [43,44].

***Metabolic Diversification:*** Extremophiles exhibit diverse metabolic pathways that enable them to utilize unconventional energy sources and tolerate extreme conditions [45]. For instance, some extremophiles can metabolize inorganic compounds, such as sulfur, to obtain energy [46,47], demonstrating metabolic flexibility that supports their colonization of extreme environments [48]. Enzymes like sulfide quinone oxidoreductase (SQR) are important for the oxidation of sulfur compounds, enabling acidophilic and thermophilic organisms like *Sulfolobus acidocaldarius* to derive energy from inorganic sulfur sources in hot, acidic environments [49]. SQR enzymes have various biotechnological applications: they can be used in bioremediation processes to detoxify and convert sulfur compounds in polluted environments, aiding in the cleanup of contaminated sites by facilitating the reduction in sulfur-containing compounds [50]. Further, SQR enzymes can be utilized in bioenergy production processes, particularly in converting sulfur compounds into biofuels or other energy carriers [51]. Their role in electron transfer and redox reactions is crucial for efficient energy conversion. SQR enzymes are valuable in various industrial processes due to their ability to catalyze redox reactions involving quinones and sulfur compounds. This includes applications in the synthesis of chemicals [52]. Different oxidoreductases are being explored for their potential in biocatalysis, particularly in the synthesis of complex organic molecules. Their ability to perform specific redox reactions makes them valuable in synthetic chemistry.

***Symbiotic Relationships:*** Extremophiles sometimes engage in symbiotic relationships, where two or more species collaborate to support each other’s survival in extreme conditions. These interactions can drive co-evolution, resulting in the further diversification of extremophiles. Proteins play a critical role in facilitating these symbiotic relationships, enabling extremophiles to thrive in harsh environments by interacting beneficially with other organisms. One example is nitrogenases, an enzyme that is essential in the symbiosis between extremophiles such as diazotrophic bacteria and plants or algae. Nitrogenase is essential for fixing atmospheric nitrogen into a usable form for the host. This is crucial in extreme environments where nitrogen availability is limited [53]. In agricultural biotechnology, nitrogenases are used to enhance nitrogen fixation in crops, reducing the reliance on synthetic nitrogen fertilizers. Approaches include engineering plants or microorganisms to express nitrogenase or developing microbial inoculants that harbor nitrogen-fixing bacteria, thus improving soil fertility and crop yields [54,55].

Other examples are peroxidases and superoxide dismutases, which help extremophiles manage oxidative stress. In symbiotic relationships, particularly in environments with high radiation or reactive oxygen species, extremophiles often have enhanced peroxidase and superoxide dismutase activities; these enzymes play a protective role for both symbionts [56]. Both enzymes have also been used in biotechnological applications in the field of bioremediation to degrade environmental pollutants including dyes and organic contaminants; bio-electrochemistry to develop biosensors for detecting environmental toxins; biomedical applications; and biopharmaceuticals [57]. These examples illustrate the diverse and impactful roles of peroxidases and superoxide dismutases from extremophiles in various biotechnological fields.

Isolation and Speciation: Extreme environments often serve as isolated niches, limiting gene flow between populations and promoting speciation. Geographic and ecological isolation can result in the evolution of distinct species adapted to specific extreme conditions [58]. Within this context, heat shock proteins (HSPs), such as HSP70 and HSP90, have evolved as crucial adaptive mechanisms. These proteins protect cells from thermal stress by assisting in protein folding and preventing aggregation. In extremophiles, HSPs help them survive extreme temperatures [59]. Both HSPs are widely used to improve the expression and stability of recombinant proteins in various host systems [60]. Their chaperone activity ensures the proper folding of proteins, which is crucial for producing functional proteins in biotechnological applications [61].

***Stress Response Mechanisms:*** Extremophiles often possess specialized stress response mechanisms that protect them from the damage caused by extreme conditions. For example, extremophiles in high-radiation environments may have efficient DNA repair systems or protective pigments.

Interestingly, in most cases, a few proteins are sufficient to guarantee the survival and thriving of extremophilic organisms in extreme habitats [62]. This might be because one or two dominant stress factors such as salt concentration, radiation, heat, or others often characterize extreme environments. These factors can frequently be neutralized by the biofunctionality of a single extremoprotein, allowing the cell or organism to remain viable. Therefore, extreme habitats can be seen as crucial drivers of evolution and adaption.

## 3. Extreme Habitat as Key Decision Maker

Extremophiles are primarily classified based on the specific extreme conditions prevalent in their habitats, rather than the type of organism (Table 2).

While prokaryotic organisms can be found in nearly any extreme environment, it is important to note that the study of eukaryotic extremophiles is an evolving field. Ongoing discoveries in this area continue to broaden our understanding of life’s adaptability to extreme conditions [6].

Extremophilic eukaryotes can be either monocellular (unicellular) or multicellular [63], encompassing a diverse array of organisms. These include single-celled entities, such as extremophilic protists and fungi, as well as multicellular species, including extremophilic algae, extremotolerant fungi, fishes, insects, and even mammals. In the following section, we delve into prominent and representative higher organisms that have evolved in response to particular environmental categories.

At the forefront of coping with hyperthermic conditions, unicellular eukaryotes thriving in extreme environments are not uncommon, and include certain species of archaea and protists. Thermophilic protists, for instance, exhibit remarkable adaptability to extremely high-temperature environments, such as hot springs [64]. Furthermore, certain unicellular extremophilic fungi and algae are capable of surviving and growing in highly acidic conditions, like those found in acid mine drainage sites [65]. Examples include the acidophilic algae *Euglena mutabilis* and various acidophilic fungi [66]. In general, eukaryotes have a lower temperature tolerance compared to prokaryotes or archaea, with the highest reported temperature being 62 °C, and most being unable to grow above 50 °C [1].

Among extremophilic eukaryotes, algae species hold particular significance. They exhibit autotrophic or mixotrophic behavior, displaying high diversity in metabolism and habitats, and various species produce organic material in water or soil [67]. Originating from endosymbiosis between a cyanobacteria and an ancient eukaryotic organism over a billion years ago, algae paved the way for oxygen production and chloroplast establishment [68]. Red and green algae, which evolved from these ancestral eukaryotic algae, along with a range of other extremotolerant organisms, play a remarkable ecological and evolutionary importance [69]. Furthermore, unicellular algae serve as excellent model systems for experimental simulations and applied studies in laboratory settings [70]. Unicellular algal cultures react by producing relatively homogeneous populations compared to other cell types under stable environmental conditions, such as light, nutrient concentration, etc. Their rapid growth—particularly compared to plant cells—makes them well-suited for large-scale bioproduct formation, including pigments, nutraceuticals, and even biofuel [71].

The thermoacidophilic red algae *Galdieria sulphuraria* thrives in highly acidic environments (pH 0) and temperatures up to 56 °C. This organism employs a combination of photosynthetic and heterotrophic growth, utilizing various carbon sources while exhibiting tolerance to high levels of activated oxygen and heavy metals. Depending on the strain, *G. sulphuraria* possesses a compact genome (13.1 to 16.0 Mb) distributed across 72 to 73 chromosomes, suggesting a robust mechanism for adaptability through a large number of chromosomes relative to its genome size [72]. The exceptional adaptability and resilience of *G. sulphuraria* underscores the potential of unicellular eukaryotes to address environmental challenges such as acid mine drainage (AMD), where mining activities lead to major hydrological and geochemical issues and reduced biodiversity [73]. AMDs can contaminate soil, surfaces, and ground sediments by high concentrations of sulfates and heavy metals in water. Unicellular eukaryotes, including algae such as *Chlamydomonas* or *Euglena*, alongside protozoa and multicellular protists, therefore demonstrate biotechnological potential for environmental remediation [74].

The reduction in biodiversity and selection pressure are not only potential outcomes of human-caused ecological changes. Climate change-induced abiotic alterations, such as those leading to frost, heat, and increased salinity shock, can also impact the community composition of unicellular eukaryotes (plankton) in hydro-ecosystems [75]. Experimental simulations conducted across different sea regions worldwide, e.g., warm and cold seas, revealed dynamic changes in plankton community composition in response to heat, consistent with findings from previous studies [76]. While increased tolerance to salinity could not reconfirm previous assumptions about lowered biodiversity [77], it appears that high diversity within the local community composition of plankton is only facilitating the enrichment of high-salinity-tolerant members, provided they are present in the population [75], rather than being driven by adaptability alone. Noteworthy, increased temperature and salinity have served as two long-standing stressors over millions of years of evolution, shaping different local planktic communities. In the case of heavy metal exposure, dynamic tolerance has been observed in the unicellular eukaryote protozoa *Tetrahymena thermophila* to titaniumoxide (TiO_2_) particles, as demonstrated by proteomics studies [78]. Various metabolic pathways were found to be significantly altered in response to different TiO2 concentrations, with no apparent toxic effects even at high amounts of heavy metals. These findings highlight the physiological adaptability within one single species and suggest promising applications for heavy metal pollution sanitation and waste management through the internalization of TiO_2_ particles by phagocytic activity [79].

Another notable example of a unicellular extremophile is the red alga *C. merolae,* recognized as one of the first fully sequenced algal genomes and the one complete eukaryotic genome assembled without gaps [80,81,82]. Its nuclear genome, in terms of the number of protein-coding genes and RNA genes, is comparable to biotechnologically established yeasts such as *Saccharomyces cerevisiae*. *C. merolae* harbors genes crucial for photosynthesis and chloroplast biogenesis and regulation, making it a promising host cell candidate for the production of bioproducts like pigments, feed, and proteins [83]. This extremophile grows in acidic seawater-nutrient media, enabling nonsterile fermentations with a low risk of contamination [83]. Various transformation techniques have been developed or are currently under development to enhance DNA introduction into *C. merolae,* driven by the increasing demand for low-cost protein-based pharmaceuticals. This includes vaccine production within biomanufacturing platforms, which represents a huge challenge, predominantly for mucosal vaccines [84]. Photosynthetic microalgae such as *Chlamydomonas, Dunaliella, Chlorella*, *Haematococcus*, and *Spirulina* are also being actively explored as efficient host cells for recombinant proteins expression, offering economical and biotechnological advantages over other biofactories [85]. Key benefits for their use in biotechnological processes include the authentical post-translational modifications, biosafety (free from human pathogenic viruses or virus-like particles), high growth rates, availability of genetic engineering tools, and high biosynthetic capacities [77]. In summary, research on unicellular extremophilic algae is paving the way for innovative advancements in the competitive field of biotechnological manufacturing of complex molecules. Additionally, it enhances our understanding of biology adaptation systems to extreme environmental conditions driven by climate change.

Fungi, long recognized for their diverse roles in ecological processes and biotechnological applications, have also drawn researchers’ attention due to their remarkable resilience in extreme environments. These exceptional organisms, referred to as fungal extremophiles, have been discovered in a wide range of extreme habitats from the desolate expanses of hypersaline environments to the scorching temperatures of geothermal springs. In response to the harsh challenges these environments impose, fungal extremophiles have evolved a range of unique survival strategies at the cellular, physiological, and molecular levels—many of which remain only partially understood. For instance, acidophilic fungi inhabit environments with extremely low pH levels, such as acid mine drainage sites. These organisms have developed robust mechanisms for maintaining intracellular pH homeostasis, including proton-pumping ATPases that extrude excess protons. Additionally, osmolytes like trehalose and membrane lipids contribute to their adaptation to acidic conditions [86]. They also produce specialized enzymes that function optimally in low pH environments, enabling them to access nutrients unavailable to most other organisms [87].

Among halophilic fungi, the rare obligate species *Aspergillus atacamensis* exhibits remarkable tolerance to both chaotropic (MgCl_2_, LiCl, CaCl_2_, and glycerol) and kosmotropic NaCl, KCl, and sorbitol) agents. The metabolic versatility of *A. atacamensis* emphasizes its potential as a promising candidate for bioremediation or biotechnological purposes [88]. While fungi generally prefer neutral or slightly acidic pH conditions, certain species can also thrive in highly alkaline environments. These fungi are capable of producing enzymes—such as amylases and proteases—that function in alkaline conditions, in pH ranges of 8 to 11, making them valuable in industries like food and textiles. Although some research has explored the signaling pathways involved in alkaline pH tolerance, the mechanisms through which alkali-tolerant fungi maintain intracellular homeostasis and the factors enabling their extracellular enzymes to remain active in alkaline conditions remain largely unexplored [89]. Additionally, strategies like the accumulation of compatible solutes to mitigate osmotic stress are evident in other halophilic fungi, including species of Wallemia [90]. These fungi thrive in hypersaline environments such as saline lakes and salt mines. Their specialized cellular machinery ensures the continuity of essential biological processes despite high salt concentrations, further highlighting their exceptional adaptability.

Among the most renowned multicellular extremophiles are tardigrades, also known as “Water Bears”. These microscopic water-dwelling animals exhibit remarkable resilience to extreme conditions, including extreme temperatures, radiation, and desiccation [91]. Despite being multicellular, tardigrades consist of a relatively small number of cells and serve as model organisms akin to *Caenorhabditis elegans* or fruit flies, offering insights into biological mechanisms, and exhibiting an evolutionary library for coping with diverse extreme conditions, applicable in biotechnological contexts [92]. Recent research highlights the role of three protein families in tardigrades: Cytoplasmic-, Secreted- and Mitochondrial-Abundant Heat Soluble (CAHS, SAHS, and MAHS) proteins. These proteins, collectively referred to as tardigrade disordered proteins (TDPs), are not found in other organisms [93]. Remarkably, TDPs lack a stable three-dimensional structure, which may allow them to adopt different conformations in the absence of water molecules, such as during freezing, desiccation, or exposure to irradiation [94].

Additionally, certain species of some extremophilic insects, particularly those from the order *Coleoptera* and the family *Carabidae*, have adapted to survive in extreme environments such as deserts with high temperatures and low moisture levels, as well as near volcanic vents. These insects have evolved various adaptations to conserve water and regulate their body temperature. The extensive monitoring of *Coleoptera* beetles in Kunashir Island of the Kuril archipelago, Russia, has revealed increasing biodiversity over some decades. Although much of these data are still unpublished, ongoing studies are trying to provide a clearer understanding of these organisms and their adaptability processes. Typically, higher animals exhibit a lower tolerance to extreme environments compared to some lower extremophiles like archaea or fungi; however, *Coleoptera* sp. and *Carabidae* sp. demonstrate a remarkable combination of resistance to desiccation, high temperatures, and acidic conditions, making them noteworthy among multicellular extremophiles [95].

Higher animals rarely thrive in extreme environments compared to microorganisms, monocytic plants, and fungi. However, certain species, such as the African lungfish *Protopterus annectes*, demonstrate remarkable adaptations. This ancient fish, considered a living ancestor of tetrapods, can endure prolonged drought due to specific branchial proteins, Aquapurine 1 and 3. These proteins enable it to withstand desiccation and rehydration stresses [96]. Similarly, Emperor penguins (*Aptenodytes foresteri*) exhibit interesting adaptations. They dive to depths of up to 500 m, deeper than any other bird, to hunt their food and cope with water pressure by evolving high-affinity hemoglobin and various physiological strategies [97]. Another example of an extremophilic animal is the vampire squid (*Vampyroteuthis infernalis*), which inhabits depth of up to 1000 m in the deep sea, where no light penetrates and oxygen levels are extremely low, through highly adaptive strategies, including an exceptionally low metabolic rate and a detritivorous trophic strategy [98]. A lowered metabolism appears to be a key survival strategy in extreme environments, whether in the deep sea or hot and arid desert.

In summary, the expression of specific proteins plays a dominant role in support for the extremophilic character of organisms, complemented by smart physiological strategies and the production of low-molecular-weight compounds like non-canonical amino acids or subunits for biodegradable polymers.

## 4. Future Directions

The field of extremophilic research is poised to advance through several exciting avenues. One key area of focus will be the discovery and characterization of novel extremophiles from previously unexplored extreme environments, such as deep-sea hydrothermal vents, acidic hot springs, and polar ice caps. Emerging genomic and metagenomic technologies are expected to play a crucial role in uncovering the genetic and metabolic pathways that enable these organisms to thrive in such harsh conditions. Additionally, there is growing interest in the biotechnological potential of extremophiles, including the development of extremozymes for industrial applications, bioremediation of contaminated environments, and the synthesis of novel bioactive compounds. Understanding the mechanisms of extremophilic adaptation could also provide valuable insights into the origins of life on Earth and the potential for life on other planets. As interdisciplinary collaborations expand, integrating microbiology, molecular biology, bioinformatics, and environmental science, the field of extremophilic research is set to progress further, offering new perspectives on the resilience and versatility of life while driving innovative biotechnological developments.

## 5. Conclusions

Extremophilic organisms, which thrive in extreme environments such as hot springs, deep-sea hydrothermal vents, and hypersaline bodies of water, have greatly expanded our understanding of life’s resilience and adaptability. Their existence challenges traditional notions about the boundaries of biological activity and broadens the perspective on potential habitats for life on Earth and beyond. Proteins are central to enabling these organisms to survive and function under such harsh conditions, making extremophiles invaluable for studying adaptive evolution and the origins of life on Earth. Studies have shown that some extremophiles, particularly hyperthermophiles, are closely related to the universal ancestors of all life, providing valuable insights into early Earth’s conditions and the evolutionary processes that shaped life.

Proteins, such as heat shock proteins and DNA protection proteins, found in these organisms, play a pivotal role in extremophile survival, offering clues about the molecular mechanisms of early life. Additionally, extremophiles have unique proteins and enzymes, known as extremozymes, which remain stable and functional under extreme conditions. These extremozymes have found diverse applications in molecular biology, industrial processes, bioremediation, biodegradation, and biomining. For instance, thermoresistant enzymes from extremophiles have been instrumental in DNA analytics and other biotechnological processes. Proteins such as the Taq polymerase, used in PCR, and sulfur-oxidizing enzymes, used in bioremediation, highlight the practical applications of extremophilic proteins. Furthermore, extremophiles often engage in symbiotic relationships and exhibit distinctive metabolic pathways, allowing them to utilize unusual energy sources and thrive in extreme environments. These interactions and metabolic flexibility are often mediated by specialized proteins, such as nitrogenases in nitrogen-fixing bacteria and peroxidases in organisms managing oxidative stress. These proteins contribute to the ability of extremophiles to colonize and influence their habitats.

Extremophiles represent a reservoir of genetic diversity, offering genes and proteins essential for adaptation to extreme conditions. This genetic diversity has positive ecological and global implications, including potential applications in food and biotech industries, wastewater treatment, and the development of sustainable biotechnological tools. Proteins that enable extremophiles to metabolize unusual energy sources or tolerate extreme conditions are particularly valuable for such innovations.

In conclusion, the study of extremophiles not only deepens our understanding of life’s adaptability and evolution but also holds significant potential for biotechnological advancements and the search for extraterrestrial life. At the core of these discoveries are proteins, which provide the molecular tools that empower extremophiles to thrive in some of the most inhospitable environments on the planet.

## Figures and Tables

**Table 1:** Description of extreme conditions and the associated biological stress burden.

Type of Extreme Condition	Description	Biological Consequences
Alkaline or acidic environment	Natural habitats with a pH above 9, like alkaline/soda lakes, limestone caves, and some hot springs, or with a pH under 5, such as volcanic lakes, acidic wetlands, streams, and soil and mine drainage, which are extremely acidic or basic either persistently, periodically, or for short periods.	Protein denaturation, cell membrane damage, enzyme inactivation, disruption of internal pH balance, and altered metabolic processes.
Cold	Habitats periodically or consistently below −17 °C either persistently, with regular frequency, or for short periods, like mountains, polar sites, and deep oceans.	Cell membrane damage, intracellular ice formation, dehydration, enzymatic inhibition and cellular damage, and cold adaptation.
Hot	Broadly defined, these habitats experience temperatures exceeding 40 °C either constantly, periodically, or for protracted periods. Examples include volcanic regions and geothermal streams.	Dehydration, cell membrane damage, protein denaturation, DNA denaturation, enzyme deactivation, and disruption of biological processes.
Hypersaline	Environments with salt concentrations greater than that of seawater, i.e., >3.5%, including salt lakes and mines.	Osmotic stress, shrinkage of the cell, desiccation, and enzyme inactivation.
High pressure	Habitats exposed to extreme hydrostatic pressure, such as ocean depths beyond 2000 m and deep lakes.	Cellular compression, enzyme inactivation, membrane disruption, possible DNA and protein denaturation, and cellular adaptation.
Radiation	Environments with background radiation levels exceeding the natural average annual exposure of approximatively 2.4 mSv (240 mrem).	DNA damage, cell death, carcinogenesis, cell dysfunction, and cell cycle arrest.
Absence of water	Habitats lacking free water whether persistently, periodically, or for short periods, including hot and cold desert environments, and some endolithic habitats.	Dehydration, protein denaturation, cell dysfunction, impaired cellular communication and functions, metabolic inactivity, growth arrest, and death.
Absence of light	Regions inaccessible to sunlight, like deep ocean environments and caves.	Reduced energy production, retarded biological rhythms, dependency on alternative energy source, reduced biodiversity, and increased adaptation and specialization.
Absence of oxygen	Habitats lacking free oxygen—whether persistently, periodically, or for protracted periods, including habitats within deeper sediments.	Oxygen deprivation, cellular damage and death. Development of semi- or full anaerobe metabolism.
Absence of nutrients	Areas on Earth that are nutrient-poor, such as the open ocean, deserts, and high-altitude regions.	Nutrient deficiency and energy depletion, organ dysfunction, and damages leading to death or growth arrest.
Human-made extreme environment	Anthropogenically affected habitats, including waste depots, mine tailings, oil-contaminated habitats, and areas polluted by heavy metals or organic compounds.	Severe cellular and tissue damage.

**Table 2:** Classification of extremophilic organisms based on environmental conditions.

Type of Extremophile	Type of Environment	Taxonomic Families of Extremophiles
Psychrophile	Cold environment	Mostly bacteria, archaea, and eukaryotes (algae)
Thermophile and acidophilic thermophile	Hot or hot acidic environment	Mostly bacteria, archaea, and rarely eukaryotes (fungi and algae)
Halophile or osmophile	High salt/high sugar	Mostly bacteria, archaea, and rarely eukaryotes (fungi and algae)
Acidophile	Acidic environment	Mostly bacteria, archaea, and eukaryotes (algae)
Alkaliphile	Alkaline environment	Mostly bacteria, archaea, and eukaryotes (black fungi and algae)
Barophile or Piezophile	High-pressure environment	Mostly bacteria, archaea, and rarely eukaryotes (single cell protists, deep-sea fish, and invertebrates)
Xerophile	Dry environment	Mostly bacteria, archaea, and eukaryotes (fungi)
Radiotolerant	High level of radiating environment	Mostly bacteria, archaea, and eukaryotes (fungi)
Endolithic extremophile	Rocky environment	Mostly bacteria, archaea, and eukaryotes (black fungi, lichens, and algae)

## Data Availability

Not applicable.
